# High-Quality Assembly of the Apple Fungal Pathogen *Marssonina coronaria* Genome and Functional Analysis of Candidate Effectors

**DOI:** 10.3390/plants14111638

**Published:** 2025-05-27

**Authors:** Huiting Guo, Yicong Fu, Lichi Zhong, Qiang Cheng

**Affiliations:** State Key Laboratory of Tree Genetics and Breeding, Co-Innovation Center for Sustainable Forestry in Southern China, Nanjing Forestry University, Nanjing 210037, China; htinguo125@163.com (H.G.); wasd5123333@163.com (Y.F.); zlc572040483@163.com (L.Z.)

**Keywords:** *Marssonina coronaria*, genome, effector, plant immunity

## Abstract

*Marssonina coronaria* is the causal agent of apple blotch, which poses a significant threat to apple production worldwide. Here, Illumina and Oxford Nanopore sequencing were combined to generate a high-quality *M. coronaria* YL1 genome assembly (54.5 Mb, 23 contigs). Based on genome annotation, 97 candidate effector proteins (CEPs) were identified, and 61 CEPs were successfully cloned for functional analysis. Transient expression assays in *Nicotiana benthamiana* revealed that eight CEPs significantly suppressed BAX-induced cell death, with McCEP12, McCEP23, McCEP24, and McCEP52 concurrently inhibiting flg22-triggered reactive oxygen species bursts. Two signal peptide-dependent cell death-inducing effectors were identified: McNLP1, containing an NPP1 domain, and McCEP3. McCEP3 exhibited evolutionary conservation within Ascomycota, with its homologous gene *VmMcCEP3* from *Valsa mali* inducing cell death in *N. benthamiana*. McEP03-triggered cell death was independent of BAK1/SOBIR1 receptor kinases. This study provides a high-quality genomic resource for *M. coronaria* and sheds light on the mechanisms by which its CEPs modulate host immunity, offering new insights into the molecular interactions between the pathogen and its host.

## 1. Introduction

Apple blotch, caused by *Marssonina coronaria* (teleomorph *Diplocarpon mali* and *Diplocarpon coronariae*), is a devastating fungal disease that poses a significant threat to apple orchards worldwide. It affects nearly all major apple cultivars, leading to premature defoliation, reduced tree vigor, and substantial declines in fruit yields [1,2]. *Marssonina coronaria* follows a hemibiotrophic lifestyle, initially colonizing apple leaves asymptomatically by forming intercellular hyphae and haustoria within host tissues. However, in the later stages of infection, extensive host cell collapse becomes evident, contributing to severe disease symptoms [3].

Successful colonization by hemibiotrophic pathogens during their biotrophic stages requires evasion or suppression of the host immune system [4]. The first layer of plant immunity, known as pattern-triggered immunity (PTI), is initiated when pattern recognition receptors (PRRs) on the plant cell surface detect conserved microbial-associated molecular patterns [5]. To overcome PTI, pathogenic microorganisms secrete effector proteins that interfere with host immune responses.

Similar PTI suppression strategies have been identified in fungal and oomycete pathogens. For example, the conserved fungal effector protein NIS1, including those from *Colletotrichum orbiculare* and *Magnaporthe oryzae*, inhibits the kinase activities of BAK1 and Botrytis-induced kinase 1 in *Nicotiana benthamiana*, leading to the significant suppression of PTI responses [6]. Similarly, a high-throughput functional screen of 33 RXLR effectors from the oomycete pathogen *Phytophthora infestans* identified eight effectors capable of suppressing flg22-induced PTI responses in tomato protoplasts [7].

During the transition from the biotrophic to the necrotrophic phase, hemibiotrophic pathogens often upregulate specific effector proteins that promote host cell death and facilitate nutrient acquisition. Some of these effectors exhibit toxin-like properties and are believed to play critical roles in enabling the necrotrophic stage of infection. For instance, necrosis-inducing protein effectors from *C. orbiculare* can trigger cell death in host tissues [8]. Similarly, necrosis- and ethylene-inducing peptide 1-like proteins (NLPs), a conserved family of cytotoxic effectors found in many fungal and oomycete pathogens, can induce immune responses or direct necrosis in dicot plants [9]. Another example is the effector MC69 from *M. oryzae*, which is required for successful colonization during the necrotrophic phase by promoting host cell death [10]. These findings highlight the diversity of necrosis-inducing effectors and their importance in the pathogenic strategies of hemibiotrophic fungi.

To date, more than 20 species of plant-pathogenic fungi within the *Marssonina* genus have been reported [11]. Among them, *M. coronaria*, *M. rosae*, and *M. brunnea* are particularly noteworthy because they cause severe black spot disease in apples, roses, and poplars, respectively [12,13]. Both *M. rosae* and *M. brunnea* follow a hemibiotrophic infection strategy, and functional studies on their effectors have been conducted. Yang et al. (2024) identified the *M. rosae* effector MrSEP43 using a BAX-triggered programmed cell death (BT-PCD) suppression assay, revealing its role in targeting the rose orphan protein RcBROG to suppress host immunity [14]. Similarly, Qian et al. (2022) identified four Common in Fungal Extracellular Membrane (CFEM) proteins in *M. brunnea* that suppressed BAX- and/or INF1-induced cell death and enhanced *Fusarium proliferatum* infection in *N. benthamiana* [15]. An extensively expanded IGY effector family has also been identified in *M. brunnea*, and one effector triggers cell death in hybrid poplar as assessed by transient expression assays [16]. In contrast, functional characterizations of *M. coronaria* effectors remain limited, underscoring the need for further research to elucidate their roles in pathogenicity.

In this study, Illumina short reads and Oxford Nanopore long read sequencing were combined to generate a high-quality genome assembly of the *M. coronaria* YL1 strain and to identify predicted genes encoding candidate effector proteins (CEPs). High-throughput functional assays of YL1 CEPs were conducted in *N. benthamiana*, providing valuable insights into the pathogenic mechanisms of *M. coronaria*.

## 2. Results

### 2.1. Marssonina coronaria Assembly and Candidate Effector Prediction

The genome assembly of *M. coronaria* YL1 was generated using a hybrid sequencing approach, combining Illumina short reads (6.66 Gb) and Oxford Nanopore Technologies (ONT) long reads (11.5 Gb). The final assembly comprised 23 contigs, with a genome size of 54.5 Mb and a GC content of 44.08%. The estimated sequencing coverages were 207.14× for ONT and 720.01× for Illumina. A BUSCO analysis revealed that 99.3% of the expected single-copy orthologs were present, indicating a high level of completeness. To further assess the assembly quality, Illumina reads were mapped back to the genome, achieving a 99.85% alignment rate. Additionally, RNA sequencing (RNA-seq) data from mycelia at multiple growth stages were incorporated to enhance gene prediction accuracy, leading to the annotation of 8069 protein-coding sequences (CDSs) with an average gene length of 1495 bp, accounting for 22.1% of the total genome. A BLASTP analysis (E-value < 1 × 10^−2^) comparing the YL1 proteome with those of previously reported *M. coronaria* strains NL1 [17] and DC1_JKI [18] identified 297 singleton proteins unique to YL1. The assembled and annotated genome sequences of YL1 have been deposited in GenBank (accession number ASM3703951) and are shown in Table 1.

Using the criteria of the presence of an N-terminal signal peptide, a length of ≤300 amino acids, the absence of transmembrane domains, and a cysteine-rich composition (containing ≥4 cysteine residues), 97 proteins were identified as candidate effector factors (Table S1). Among them, six proteins were unique to the YL1 strain, having no homologs among the predicted proteins from the other *M. coronaria* strains or in the NCBI non-redundant protein database (E-value < 0.05).

### 2.2. Screening of Effectors Suppressing BT-PCD

To investigate the roles of candidate effectors during *M. coronaria* infection, total RNA was extracted from inoculated apple leaves at 4 days post-infiltration (dpi) and used for complementary DNA (cDNA) synthesis. In total, 97 cysteine-rich effector genes were cloned into the potato virus X (PVX) vector for functional analyses. Sanger sequencing confirmed that the introns of 47 effector genes were successfully spliced out, indicating active transcription, and 14 with predicted intronless CDSs were also successfully cloned. In total, 61 effector CDS-PVX constructs were generated for further characterization (Table S2).

**Figure 1:**
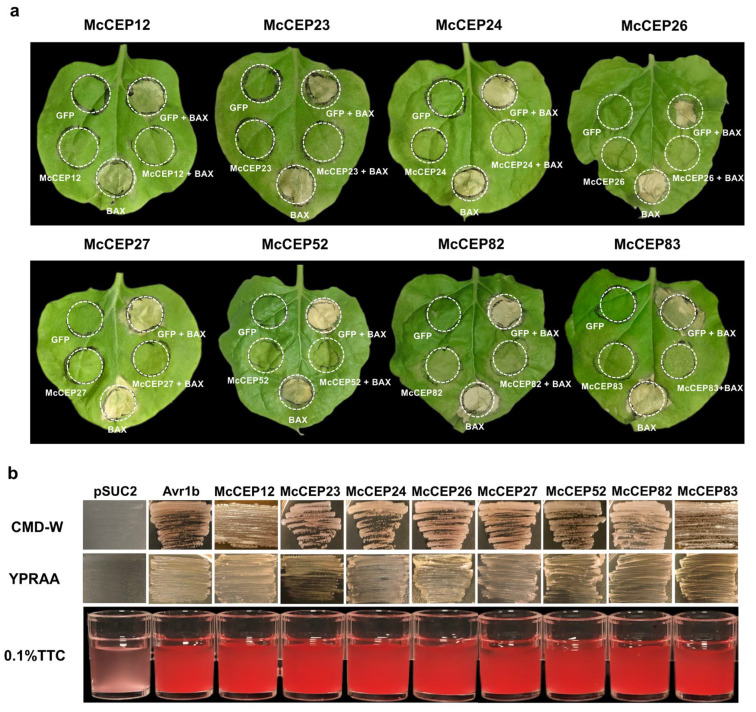
Functional characterization of candidate effectors from *M. coronaria* in *N. benthamiana*. (**a**) Suppression of BT-PCD by candidate effectors. *Agrobacterium tumefaciens* strains harboring PVX constructs expressing candidate effectors or BAX were sequentially infiltrated into 4–5-week-old *N. benthamiana* leaves. Cell death symptoms were assessed at 5 days post-infiltration (dpi). PVX-eGFP served as a negative control. (**b**) Validation of predicted signal peptides from BT-PCD-suppressing effectors using the yeast invertase secretion assay. Yeast transformants expressing signal peptides were tested for growth on CMD-W (complete medium lacking tryptophan) and YPRAA (yeast extract–peptone–raffinose–antimycin A) plates. Invertase activity was confirmed by TTC (triphenyl tetrazolium chloride) staining, where red-colored TPF (triphenyl formazan) indicated successful secretion. The Avr1b signal peptide was used as a positive control, and pSUC2 without an insert was the negative control.

To assess their abilities to modulate plant cell death, these constructs were transiently expressed in *N. benthamiana* via *Agrobacterium*-mediated transformation, followed by co-expression with BAX. Eight effectors—McCEP12, McCEP23, McCEP24, McCEP26, McCEP27, McCEP52, McCEP82, and McCEP83—effectively suppressed BT-PCD, as evidenced by the absence of visible cell death symptoms compared with the eGFP control (Figure 1a). An InterProScan analysis indicated that six of these BT-PCD-suppressing effectors lacked predicted conserved domains, whereas McCEP52 contained a glycoside hydrolase domain (PF00232), and McCEP82 harbored a cell wall protein domain (PF01473).

The secretion capabilities of the effector signal peptides were confirmed using a yeast invertase secretion assay. The signal peptides of all the BT-PCD-suppressing effectors, along with those of Avr1b (used as the positive control), successfully complemented the invertase secretion defect in *Saccharomyces cerevisiae* YTK12, enabling yeast growth on the YPRAA medium. Furthermore, enzymatic activity assays validated their secretion functionalities. Yeast strains expressing invertase fused with these signal peptides converted 2,3,5-triphenyl-2H-tetrazolium chloride (TTC) into insoluble red-colored formazan (TPF) (Figure 1b).

### 2.3. Screening of Effectors Inhibiting flg22-Induced Reactive Oxygen Species (ROS) Bursts

**Figure 2:**
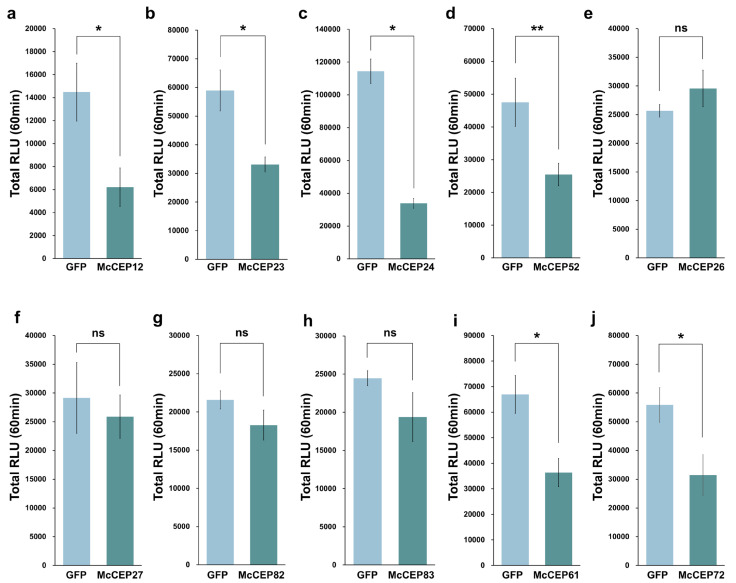
Suppression of flg22-induced ROS production by candidate effector proteins. Total ROS production (relative light units, RLU) was measured 60 min after treatment with 200 nM flg22 in *N. benthamiana* leaf disks that had been transiently expressing candidate effector proteins for 48 h. Leaf disks transiently expressing eGFP served as a negative control. Data are presented as the means ± standard errors (SEs) (*n* = 6, technical replicates). Statistical significance was assessed using a one-way analysis of variance followed by Tukey’s HSD test. Asterisks indicate statistically significant differences compared to the eGFP control (* *p* < 0.05, ** *p* < 0.01). The experiment was repeated at least three times with independent biological replicates and yielded consistent results.

To evaluate the ability of candidate effectors to suppress plant immune responses, a ROS suppression assay was performed. In total, 28 effectors were tested, including the 8 previously identified BT-PCD suppressors and 20 randomly selected effectors (Table S3). To minimize physiological differences between leaves and accurately assess the effects of candidate effectors on flg22-induced ROS production, *Agrobacterium* strains carrying PVX constructs with candidate effector CDSs (test group) or eGFP (negative control) were infiltrated into adjacent regions of *N. benthamiana* leaves. In total, four out of the eight BT-PCD-suppressing effectors (McCEP12, McCEP23, McCEP24, and McCEP52) significantly reduced ROS production compared to the eGFP control (*p* < 0.01), suggesting their potential roles in suppressing PTI (Figure 2a–d). In contrast, the remaining four BT-PCD-suppressing effectors showed no significant effects on ROS accumulation (*p* > 0.05) (Figure 2e–h). Among the 20 randomly selected effectors that did not suppress BT-PCD, only 2 effectors (McCEP61 and McCEP72) exhibited significant reductions in ROS production (*p* < 0.05) (Figure 2i,j), whereas the remaining 18 effectors showed no detectable impacts. These findings highlight that a subset of *M. coronaria* effectors capable of interfering with PTI-associated ROS bursts had considerable overlap with BT-PCD-suppressing effectors (four out of six).

### 2.4. Effectors Inducing Cell Death in N. benthamiana

**Figure 3:**
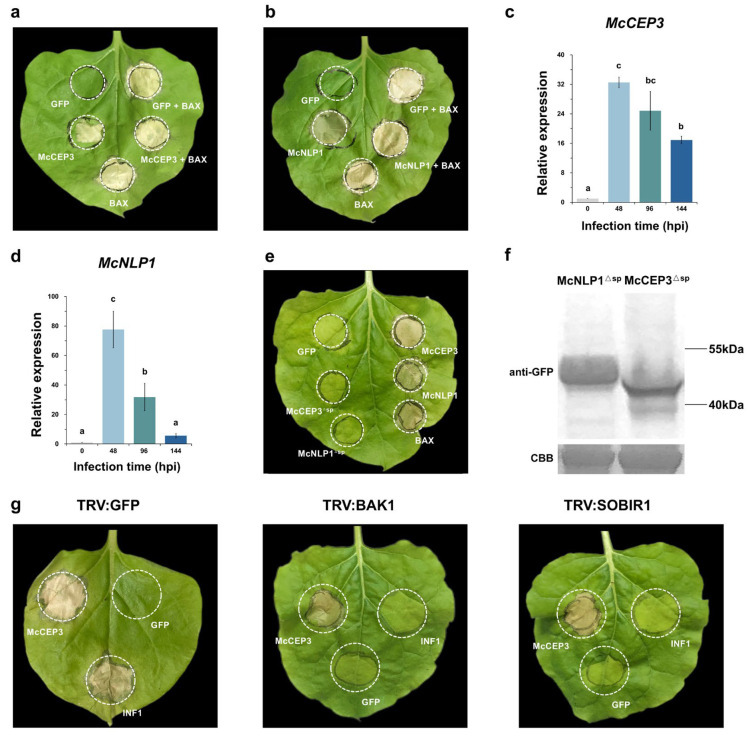
Identification of *M. coronaria* effectors that induce cell death in *N. benthamiana*. (**a**,**b**) Transient expression of McCEP3 (**a**) and McNLP1 (McCEP28) (**b**) in *N. benthamiana* using the PVX system induced cell death. PVX-eGFP was used as a negative control, and PVX-BAX served as a positive control. The experiment was repeated at least three times with consistent results, and images were taken at 5 dpi. (**c**,**d**) RT-qPCR analysis of McCEP3 and McNLP1 expression during *M. coronaria* infection in apple leaves. Data are presented as means ± standard errors (SEs) (*n* = 3, biological replicates). Different letters indicate statistically significant differences among mean values, determined by one-way analysis of variance followed by Tukey’s test (*p* < 0.05). (**e**) Transient expression of McCEP3^ΔSP^ and McNLP1^ΔSP^ (lacking their signal peptides) in 4-week-old *N. benthamiana* did not induce cell death at 5 dpi. (**f**) Western blot analysis confirming the successful accumulation of McCEP3^ΔSP^ and McNLP1^ΔSP^ in planta using an anti-GFP antibody. Protein gel staining with Coomassie Brilliant Blue was performed to ensure equal protein loading as a control. (**g**) Transient expression of McCEP3 in *N. benthamiana* plants with BAK1 or SOBIR1 silenced still resulted in cell death. INF1-induced cell death was used as a positive control, and eGFP was used as a negative control. The transient expression experiment was repeated at least three times with consistent results.

The *Agrobacterium*-mediated infiltration of *N. benthamiana* leaves with PVX constructs carrying candidate effector CDSs led to the identification of two effectors, McCEP3 and McCEP28, as inducers of cell death. At 5 dpi, both McCEP3 and McCEP28 triggered cell death symptoms comparable to those of the positive control BAX, whereas no cell death symptoms were observed in the negative control eGFP (Figure 3a,b). McCEP28 (hereafter McNLP1) contains an NLP domain (PF05630, E-value = 2 × 10^−61^) and shares high sequence identity with MbMoNLP2 (Mb2g8266) from *M. brunnea f. sp. monogermtubi* (51.4% identity) and PpNLP from *Phytophthora parasitica* (41.9% identity), both of which have been previously characterized as cell death-inducing proteins [19,20]. In contrast, McCEP3 lacks any known conserved functional domains. A qRT-PCR analysis revealed that both McCEP3 and McCEP28 were highly expressed at 48 h post-inoculation, suggesting their early activation and potential involvement in plant cell death regulation (Figure 3c,d).

To determine whether McCEP3 and McNLP1 induce cell death by targeting the plant extracellular matrix, truncated versions lacking their signal peptides (McCEP3^ΔSP^ and McNLP1^ΔSP^) were transiently expressed in *N. benthamiana* using the PVX system. Neither McCEP3^ΔSP^ nor McNLP1^ΔSP^ induced cell death, even at 6 dpi (Figure 3e). To assess whether the absence of the signal peptide affected protein accumulation, PVX expression vectors encoding C-terminal eGFP fusions for McCEP3^ΔSP^ and McNLP1^ΔSP^ were constructed and transiently expressed in *N. benthamiana*. A western blot analysis confirmed that all the proteins were properly expressed in *N. benthamiana*, regardless of the absence of their signal peptides (Figure 3f). These results indicate that extracellular secretion is essential for McCEP3- and McNLP1-induced cell death in *N. benthamiana*.

To determine whether McCEP3-induced cell death depends on BAK1 and suppressor of BIR1-1 (SOBIR1), which are key components of the PTI signaling pathway, McCEP3 was expressed in *N. benthamiana* plants in which *BAK1* and *SOBIR1* were subjected to virus-induced gene silencing (VIGS). Silencing of these genes completely abolished INF1-induced cell death, confirming their essential roles in this immune response. However, the transient expression of McCEP3 in BAK1- or SOBIR1-silenced plants still resulted in significant cell death (Figure 3g). Thus, McCEP3 may trigger PCD through a pathway distinct from the known PTI signaling cascade.

### 2.5. McCEP3 Represents a Conserved Class of Fungal Cell Death-Inducing Proteins

**Figure 4:**
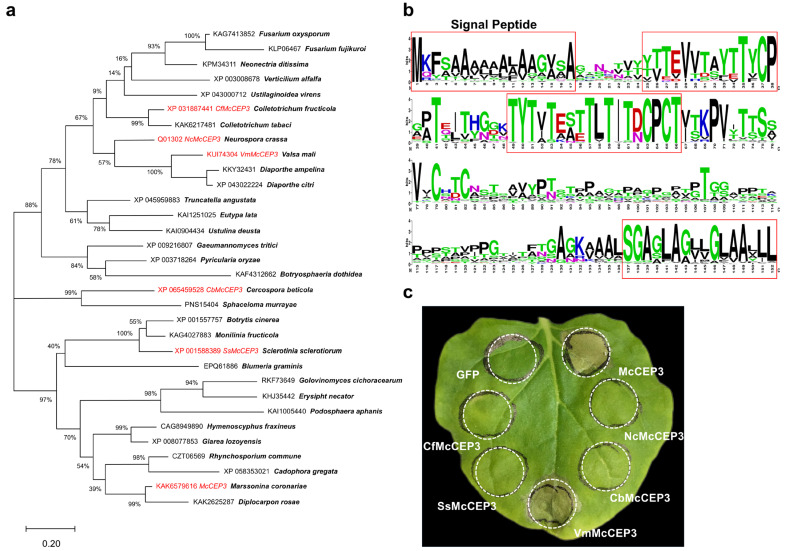
McCEP3 represents a conserved class of ascomycete proteins capable of inducing cell death in *N. benthamiana*. (**a**) A maximum likelihood phylogenetic tree was constructed based on the alignment of McCEP3 and its homologous protein sequences from multiple fungal species. Bootstrap support values (n = 1000 replicates) are shown at each node. Representative homologs from different fungal taxa are included, and genes used for transient expression in *N. benthamiana* are highlighted in red. The scale bar represents the number of substitutions per site. (**b**) WebLogo representation of sequence conservation among 505 McCEP3 homologs, with conserved regions highlighted in red boxes. (**c**) Transient expression of five McCEP3 homologs in 4-week-old *N. benthamiana* using *Agrobacterium*-mediated PVX vectors revealed that VmMcCEP3 induced visible cell death at 5 dpi, whereas the other four homologs did not. eGFP and BAX were used as negative and positive controls, respectively. The experiment was repeated at least three times with consistent results.

To investigate the conservation of McCEP3, a BLASTP search against the NCBI non-redundant database was performed. McCEP3 was widely conserved among 483 species within the Ascomycota phylum, including representatives from the Sordariomycetes, Dothideomycetes, and Leotiomycetes classes. A total of 505 homologous proteins were identified with high sequence conservation (E-value < 2 × 10^−11^), sharing more than 40% pairwise sequence identity with McCEP3 (Table S4). Among these homologs, 98% contained predicted signal peptides, and 77% had molecular lengths ranging from 150 to 200 amino acids (Table S4). Most homologs were annotated as hypothetical proteins, with only one, *CCG-6* from *Neurospora crassa*, having been functionally characterized and implicated in circadian rhythm regulation. A phylogenetic tree was constructed using representative sequences from each genus (Figure 4a). It revealed a topology that closely mirrored species taxonomic relationships. Notably, conserved motifs were observed at both the N- and C-termini (Figure 4b).

To functionally assess whether McCEP3 homologs share a conserved role, genes from five major phylogenetic branches were selected for synthesis and cloned into the PVX vector for transient expression in *N. benthamiana* (Figure 4c). Notably, the transient expression of only *Valsa mali* VmMcCEP3, which shows 57% sequence identity to McCEP3, also induced significant cell death in *N. benthamiana* leaves, suggesting that certain fungal McCEP3-like proteins have a conserved role in PCD induction (Figure 4c).

## 3. Discussion

### 3.1. Comparative Genomic Analysis of M. coronaria

*Marssonina coronaria* is a major fungal pathogen that poses a significant threat to global apple cultivation. To date, two studies have specifically reported and analyzed the *M. coronaria* genome. The first study presented the NL1 strain (50.3 Mb, 589 scaffolds, N50: 231.4 kb), assembled using Illumina sequencing [17]. More recently, another study reported a high-quality genome for DC1_JKI (51.5 Mb, 22 contigs, N50: 4.2 Mb) from Dresden, Germany, which was generated using a hybrid sequencing strategy combining Illumina short-read sequencing and Oxford Nanopore MinION long-read sequencing [18].

In this study, another high-quality genome, the YL1 strain from Yangling, China, which was also assembled using a hybrid strategy, was presented. The YL1 genome (54.5 Mb) is the largest *M. coronaria* genome reported to date, with a contig number (23) and an N50 value (4.1 Mb) comparable to those of DC1_JKI and significantly larger than those of NL1. A genome completeness assessment using a BUSCO analysis (99.3%) further confirmed that the YL1 genome has the highest assembly quality among reported *M. coronaria* genomes. In terms of genomic similarity, DC1_JKI exhibits a higher sequence similarity to NL1, with a 76.84% genome coverage under a strict sequence identity threshold (≤5% divergence), whereas YL1 covers only 66.98% of DC1_JKI. A phylogenetic analysis further supports DC1_JKI being more closely related to NL1 than YL1, with the latter representing a more distantly related lineage [18]. Furthermore, unlike previous studies that relied primarily on homology-based gene predictions, RNA-seq data from mycelia at different germination stages (2, 4, and 6 days) was incorporated as annotation hints. This approach enabled a more comprehensive identification of protein-coding genes, particularly those expressed during the infection stage. As a result, our analysis identified 297 protein-coding genes unique to the YL1 strain.

### 3.2. Effector Identification and Functional Characterization

Effectors play crucial roles in the pathogenicity of hemibiotrophic fungi. However, previous genomic studies on *M. coronaria* did not explore the functional characterization of its effectors. The relationship between BT-PCD suppression and plant immune modulation remains unclear, even though BT-PCD suppression assays in *N. benthamiana* are widely used for effector screening. Effectors identified through BT-PCD suppression assays can also inhibit plant PTI responses. For example, the *Lasiodiplodia theobromae* effector CSEP1 not only suppresses BAX-induced cell death in *N. benthamiana*, but also significantly inhibits flg22-induced ROS bursts and defense-related gene expression, thereby attenuating PTI [21]. Similarly, the *Phytophthora sojae* effector PsCRN70 suppresses both BAX- and INF1-induced cell death in *N. benthamiana* and reduces ROS accumulation, as well as defense gene expression, thereby promoting pathogen virulence [22]. Additionally, the *Plasmopara viticola* effector PvRXLR159 blocks BAX- and INF1-triggered cell death and suppresses PTI-mediated resistance against *Phytophthora capsici* [23].

In this study, eight *M. coronaria* effectors that suppressed BAX-induced cell death in *N. benthamiana* were identified, and four also inhibited flg22-induced PTI responses. This overlap suggests that some effectors target common immune regulators involved in both PTI and cell death signaling. While these findings align with previous studies that suggested functional convergence between effectors that suppress PTI and BT-PCD, the precise molecular targets of these *M. coronaria* effectors remain to be elucidated.

### 3.3. McCEP3 and the Mechanism of Cell Death Induction

In addition to suppressing cell death, two *M. coronaria* effectors that induce cell death were identified. One of them, McNLP1, belongs to the NLP family, which is widely present in oomycetes, fungi, and bacteria. NLPs are well-known cytolytic toxins that specifically target dicotyledonous plants. Their modes of action involve binding to glycosyl inositol phosphoryl ceramide sphingolipids in the plant plasma membrane and forming pores, leading to osmotic disruption and cell lysis [24]. The second effector, McCEP3, is widely distributed among ascomycetes. Among the six artificially synthesized McCEP3 homologs, one homolog from *V. mali* (a causal agent of apple canker) was also capable of inducing cell death in *N. benthamiana*. A gene expression analysis further revealed that McCEP3 shares a similar expression pattern with McNLP1, reaching its highest transcript levels during the transition from biotrophic to necrotrophic growth in infected apple leaves. Thus, McCEP3 and its homologs may represent a novel family of conserved toxic effectors in ascomycete fungi that facilitate pathogen colonization during the necrotrophic phase of infection.

To determine whether McCEP3-induced cell death relies on known PRR-mediated immune signaling, we used VIGS to knock down BAK1 and SOBIR1, two key components of the PTI signaling pathway. As expected, silencing BAK1 and SOBIR1 completely abolished INF1-induced cell death in *N. benthamiana*, confirming their essential roles in this immune response. However, McCEP3-induced cell death remained unaffected, suggesting that its mechanism of action is independent of the canonical PTI signaling pathway.

This result implies that even if McCEP3 interacts with a membrane-associated receptor, its mechanism of triggering cell death differs from known PRR-mediated immune pathways. Further studies are needed to elucidate whether McCEP3 functions as a direct toxin or activates an alternative, yet unidentified, plant immune response leading to cell death.

## 4. Materials and Methods

### 4.1. Fungal and Plant Materials, and Pathogen Inoculation

The *M. coronaria* YL1 strain was isolated in 2018 from an apple (*Malus domestica* Borkh) leaf exhibiting blotchy lesions in Yangling, Shaanxi Province, China (34.16° N, 108.05° E). The strain was cultured on potato dextrose agar (PDA) plates and incubated at 25 °C. *N. benthamiana* and apple seedlings were maintained in a greenhouse under controlled environmental conditions: 25 °C, a 12-h light/12-h dark photoperiod, and 60% relative humidity. Conidia of YL1 were harvested from PDA cultures after 10 days of incubation. The conidia were suspended in deionized water, adjusted to a final concentration of 10,000 spores/mL, and uniformly sprayed onto apple leaves. The inoculated plants were incubated under 100% humidity at 25 °C.

### 4.2. Whole-Genome Sequencing and Annotation of YL1

The genomic DNA of *M. coronaria* YL1 was extracted using the DNAsecure Plant Kit (Tiangen, Beijing, China). Long-read sequencing libraries were prepared using the SQK-LSK109 ligation kit (Oxford Nanopore Technologies, Oxford, UK) following the manufacturer’s protocol and subsequently sequenced on the Oxford Nanopore PromethION platform for real-time single-molecule sequencing. The same DNA was also used to construct sequencing libraries with the Nextera DNA Flex Library Prep Kit (Illumina, San Diego, CA, USA) and sequenced on the Illumina NovaSeq 6000 platform to generate 150-bp paired-end reads. Library preparation and sequencing were performed by Benagen (Wuhan, China). Raw genome assemblies were generated using NECAT [25], and error correction was performed with Pilon v1.23 [26] using Illumina reads. The completeness of the assembled genome was evaluated using BUSCO v4.1.2 against the fungi_odb10 dataset [27].

For the transcriptomic analysis, *M. coronaria* mycelia were collected from cultures grown on cellophane membranes placed on PDA plates at 25 °C for 2, 4, and 6 days. Total RNA was extracted using the RNAsecure Plant Kit (Tiangen), and equal amounts of RNA from the three-time points were pooled to construct a single RNA-seq library. The library was prepared using the TruSeq Stranded mRNA Library Prep Kit (Illumina) and sequenced on the Illumina NovaSeq 6000 platform, generating 150-bp paired-end reads. RNA-seq data were utilized as annotation hints for gene predictions using Braker2 [28].

Candidate effectors were identified using SignalP 5.0 [29] and TMHMM [30]. Effector candidates were selected based on the presence of a predicted signal peptide (SignalP), the absence of transmembrane helices in the mature protein (TMHMM), and a sequence length not exceeding 300 amino acids. Effector domain identification and functional annotation were conducted using InterProScan (version 5) via its web server (https://www.ebi.ac.uk/interpro/ accessed on 10 March 2023).

### 4.3. RNA Extraction and Quantitative PCR (qPCR)

Total RNA was extracted from apple leaves inoculated with the *M. coronaria* YL1 strain at time points ranging from 0 to 6 days using the RNA extraction kit (Tiangen). The cDNA was synthesized using a reverse transcription kit (Vazyme Biotech, Nanjing, China) following the manufacturer’s instructions. qPCR was conducted under the following cycling conditions: an initial denaturation at 95 °C for 30 s, followed by 40 cycles of 95 °C for 5 s, 60 °C for 30 s, and 72 °C for 30 s. The *M. coronaria EF-1α* gene served as an internal control. Relative transcription levels of target genes were calculated using the 2^−ΔΔCt^ method. Statistical significance was assessed using an unpaired *t*-test with data obtained from three biological replicates. The significance threshold was set at *p* < 0.05. The primers used for the gene expression analysis are listed in Table S5.

### 4.4. Candidate Effector Cloning and Plasmid Construction

Candidate effector CDSs were amplified from cDNA derived from apple leaves at 4 days after inoculation with the *M. coronaria* YL1 and subsequently inserted into the PVX vector pGR107 [31]. The signal peptide sequences of candidate effector proteins were cloned into the pSUC2 vector. Additionally, *BAK1* and *SOBIR1* gene fragments were amplified from *N. benthamiana* cDNA and inserted into the pTRV2 vector. All the constructs were generated using the ClonExpress Ultra One-Step Cloning Kit (Vazyme). The primers and restriction sites used for cloning are listed in Table S5. All the plasmid constructs were verified by Sanger sequencing to confirm sequence integrity.

### 4.5. Cell Death Induction and the BT-PCD Suppression Assay

The ability of candidate effector proteins to induce cell death and suppress BT-PCD was assessed in *N. benthamiana* using *Agrobacterium*-mediated transient expression. All the PVX constructs with candidate effector CDSs were introduced into *A. tumefaciens* strain GV3101 (pJIC SA_Rep). *Agrobacterium* cultures were infiltrated into the leaves of 4- to 5-week-old *N. benthamiana* plants. After 12 h, the same leaf regions were infiltrated with *Agrobacterium* carrying the BAX gene. Green fluorescent protein (GFP) was used as a negative control. Each candidate effector was tested on six leaves, and the experiment was repeated three times. At 5 dpi, leaf cell death symptoms were recorded.

### 4.6. Validation of SP Secretory Activities

The secretory functions of the predicted effector proteins’ signal peptides were assessed using the Yeast Signal Sequence Trap System [32]. The signal peptide sequences of candidate effector proteins were individually cloned into the pSUC2 vector and subsequently transformed into *S. cerevisiae* strain YTK12. Transformed yeast strains were first screened on a CMD-W medium (0.67% yeast nitrogen base without amino acids, 0.075% tryptophan dropout supplement, 2% sucrose, 0.1% glucose, and 2% agar). Positive clones were then cultured on a YPRAA medium (1% yeast extract, 2% peptone, 2% raffinose, and 2 mg/mL antimycin A) for invertase secretion assays. The pSUC2 construct containing the functional Avr1b signal peptide (*pSUC2::Avr1b*) was used as a positive control, and the empty pSUC2 vector served as a negative control.

Enzyme activity was detected using a colorimetric assay, in which TTC was reduced to insoluble TPF [33]. Transformed yeast strains were inoculated in a CMD-W liquid medium, incubated at 30 °C with shaking at 220 rpm for 24 h, and collected by centrifugation. The cell pellets were resuspended in 1 mL of 10% sucrose solution, followed by the addition of 1% TTC solution, and incubated at 30 °C for 30 min. The development of a red color was used to assess the secretory activity of each effector protein’s signal peptide.

### 4.7. Flg22-Induced ROS Suppression Assay

The suppression of flg22-induced ROS production by candidate effector proteins was assessed following the method described by Sang et al. [34]. At 48 h after infiltration with *A. tumefaciens* GV3101 (pJIC SA_Rep) carrying the PVX vector and candidate effector CDSs into the leaves of 4- to 5-week-old *N. benthamiana* plants, 0.5 cm leaf disks were excised and floated in 200 μL of sterile water in a 96-well plate overnight. The water was then replaced with 200 μL of reaction buffer containing 200 nM of flg22, 100 nM of luminol, and 10 mg/mL of horseradish peroxidase. Luminescence was recorded using a GloMax™ 96 microplate reader (Promega, Fitchburg, WI, USA) to quantify ROS production.

### 4.8. Protein Extraction and Western Blot Analysis

At 48 h post-inoculation, *N. benthamiana* leaves (approximately 1.5 cm in diameter) were harvested and homogenized in 200 μL of protein extraction buffer (125 mM Tris-HCl, pH 8.8; 1% SDS; 10% glycerol; 50 mM sodium metabisulfite), and mixed with 2× Laemmli buffer at a 4:1 volume ratio. The samples were boiled for 5 min, and the supernatant was collected and stored at −80 °C. For protein analysis, 30 μL of each sample was separated by electrophoresis using a Tris-MOPS-SDS precast gel (GenScript, Nanjing, China) under a constant current of 40 mA. Proteins were then transferred to a 0.45 μm nitrocellulose membrane at 80 V for 2 h. After transfer, the membrane was blocked with 5% BSA for 1 h, followed by incubation with an anti-GFP primary antibody overnight at 4 °C, and then with an HRP-conjugated secondary antibody for 1 h at room temperature. Target protein bands were visualized using BCIP/NBT substrate.

### 4.9. VIGS Assay

The VIGS assays were conducted in *N. benthamiana* using the tobacco rattle virus (TRV) vector system [35]. The pTRV1 and pTRV2 construct independently containing BAK1 (*pTRV2::BAK1*) and SOBIR1 (*pTRV2::SOBIR1*), respectively, were separately transformed into *A. tumefaciens* GV3101. The *A. tumefaciens* strains carrying *pTRV2::BAK1* or *pTRV2::SOBIR1* were mixed in a 1:1 ratio with GV3101 carrying pTRV1 and subsequently infiltrated into the primary leaves of 4-leaf-stage *N. benthamiana* plants. The *pTRV2::GFP* construct was used as a negative control. Each experiment was performed in three independent biological replicates, with six leaves from three individual plants per replicate.

### 4.10. Phylogenetic Tree Construction

Homologous sequences of the target proteins were retrieved from the NCBI database using BLASTP (E-value < 2 × 10^−11^). Representative sequences were selected based on taxonomic coverage (ensuring at least one sequence per genus) and sequence integrity (prioritizing full-length or near full-length sequences without missing critical domains). Multiple sequence alignments were performed using the MAFFT tool, and the phylogenetic tree was generated with the Maximum Likelihood method using the MEGA 7.0 software [36]. The reliability of the tree topology was evaluated by bootstrap resampling with 1000 replicates.

## Figures and Tables

**Table 1:** Summary of the *M. coronaria* YL1 genome assembly and annotation.

Genome Feature Annotation	Statistics
NCBI accession	PRJNA940293
Genome size (bp)	54,484,178.00
Contigs	23
N50 contig (bp)	4,071,588.00
Maximum contig length (bp)	2,368,877.30
GC content (%)	44.08
Gene number	8095
mRNA average length (bp)	2756.83
CDS average length (bp)	1495.95
Transfer RNA number	8275
Ribosomal RNA number	42,433
Small nuclear RNA number	3638

## Data Availability

The genome sequence data have been deposited in GenBank under accession number ASM3703951. The data supporting the findings of this study are available in the main text and the Supplementary Materials.

## Supplementary Materials

The following supporting information can be downloaded at: https://www.mdpi.com/article/10.3390/plants14111638/s1, Table S1: Characteristics of 97 candidate effectors from *Marssonina coronaria*; Table S2: Functional analysis of 61 *M. coronaria* effectors in *N. benthamiana*; Table S3: Suppression of flg22-induced ROS production by candidate *M. coronaria* effector proteins; Table S4: Sequence characteristics of McCEP3 homologous proteins; and Table S5: Primers used in this study.
