# Supplementary material for: High-Quality Assembly of the Apple Fungal Pathogen Marssonina coronaria Genome and Functional Analysis of Candidate Effectors
Source: Plants (Basel). 2025 May 27;14(11):1638. doi: 10.3390/plants14111638 (PMC12158172; doi:10.3390/plants14111638)
Supplement: Supplementary file 1 [file plants-14-01638-s001.zip › Supplementary Table S2-S3.pdf]

Table S2. Functional Analysis of 61 Effectors in *Nicotiana benthamiana*

**Number**	**BT-PCD Suppression / Cell Death Induction**
McCEP03	Cell Death Induction
McCEP28	Cell Death Induction
McCEP12	BT-BAX Suppression
McCEP23	BT-BAX Suppression
McCEP24	BT-BAX Suppression
McCEP26	BT-BAX Suppression
McCEP27	BT-BAX Suppression
McCEP52	BT-BAX Suppression
McCEP82	BT-BAX Suppression
McCEP83	BT-BAX Suppression
McCEP01	-
McCEP02	-
McCEP04	-
McCEP05	-
McCEP06	-
McCEP07	-
McCEP08	-
McCEP09	-
McCEP10	-
McCEP13	-
McCEP16	-
McCEP17	-
McCEP18	-
McCEP19	-
McCEP25	-
McCEP32	-
McCEP33	-
McCEP34	-
McCEP35	-
McCEP36	-
McCEP37	-
McCEP40	-
McCEP42	-
McCEP49	-
McCEP51	-
McCEP53	-
McCEP54	-
McCEP55	-
McCEP56	-
McCEP57	-

McCEP58	-
McCEP61	-
McCEP67	-
McCEP68	-
McCEP71	-
McCEP72	-
McCEP73	-
McCEP74	-
McCEP76	-
McCEP77	-
McCEP78	-
McCEP79	-
McCEP80	-
McCEP81	-
McCEP86	-
McCEP88	-
McCEP90	-
McCEP91	-
McCEP92	-
McCEP94	-
McCEP97	-

"BT-PCD Suppression" indicates that the effector can suppress BT-PCD in *N. benthamiana*."Cell Death Induction" indicates that the effector can induce cell death in *N. benthamiana*."-" indicates no detectable BT-PCD suppression or cell death induction activity in *N. benthamiana*.

Table S3. Suppression of flg22-induced ROS production by candidate effector proteins

Sample	Total RLU (60min)
GFP-McCEP12	14476
McCEP12	6208
GFP-McCEP23	58951
McCEP23	33104
GFP-McCEP24	114387
McCEP24	33877
GFP-McCEP26	25644
McCEP26	29552
GFP-McCEP27	29143
McCEP27	25890
GFP-McCEP52	47528

McCEP52	25451
GFP-McCEP82	21574
McCEP82	18274
GFP-McCEP83	24458
McCEP83	19384
GFP-McCEP05	28234
McCEP05	30888
GFP-McCEP07	30634
McCEP07	33557
GFP-McCEP08	27721
McCEP08	29340
GFP-McCEP16	25815
McCEP16	29973
GFP-McCEP32	25583
McCEP32	29060
GFP-McCEP33	38994
McCEP33	43018
GFP-McCEP35	28919
McCEP35	32569
GFP-McCEP42	41309
McCEP42	44557
GFP-McCEP55	31102
McCEP55	34062
GFP-McCEP56	28102
McCEP56	32975
GFP-McCEP61	69969
McCEP61	36352
GFP-McCEP68	30640
McCEP68	33417
GFP-McCEP72	55832
McCEP72	31436
GFP-McCEP73	31343
McCEP73	33358
GFP-McCEP74	22124

McCEP74	26775
GFP-McCEP86	33656
McCEP86	36884
GFP-McCEP88	32150
McCEP88	34111
GFP-McCEP94	23507
McCEP94	27861
GFP-McCEP97	40343
McCEP97	38358
